# On the road to Mecca: Branding discourses and national identity on coffee shop signage

**DOI:** 10.1371/journal.pone.0309829

**Published:** 2025-02-04

**Authors:** Abduljalil Nasr Hazaea, Mutahar Qassem

**Affiliations:** 1 Department of English Language Skills, Deanship of Preparatory Year, Najran University, Najran, Saudi Arabia; 2 Department of Translation, College of Languages and Translation, Najran University, Najran, Saudi Arabia; Universiti Malaya, MALAYSIA

## Abstract

Commercial branding stands as a discursive and cultural facet of the contemporary global era where competing brands construct their own identities. From a discourse perspective, a brand is discursively constructed on commercial signs. Accordingly, this study examines the interplay between coffee shop branding and national identity in Saudi Arabia. In so doing, the study investigates the competing branding discourses associated with coffee as well as the space given to national identity. To achieve this task, the study developed a conceptual framework grounded on critical discourse analysis (CDA) and linguistic landscape (LL). The data consists of 88 commercial signs of coffee shops collected by driving on a road from Najran to Mecca, Saudi Arabia. The research site was then verified through Google Maps. The data built a communicative event for an empirical mixed-method research design. CDA linguistic and multimodal toolbox was utilized. The analysis showed that three names of coffee are found on the road to Mecca: qahwa (Arabic), coffee (English), and kufi (transliteration). With these names, four discourse are in competition. For globalization, English-Arabic glocal discourse (34%), and English global discourse (8%) are competing to construct coffee branding. For national identity, Arabic local discourse (42%) and Arabic-English glocal discourse (16%) are associated with qahwa; something that gives substantial space (58%) for national identity. These findings enhance our understanding of the linguistic and multimodal dimensions of globalized spaces and their discursive construction of branding at the local scale of globalization. The study recommends further research and suggests some cultural and pedagogical implications for authorities, translation, bilingual awareness, teaching, and learning.

## Introduction

Commercial brands are everywhere, serving not only to promote the efficiency of the products they represent but also to construct and communicate identities. The study of brands has primarily been situated within the field of market research, where scholars have examined how brands influence consumer behavior and shape market trends [1–3]. This focus has provided valuable insights into the economic functions of branding, emphasizing how brands compete and succeed in the marketplace. However, there has been a significant shift in recent years as the analysis of branding moves from traditional market research into the domain of discourse studies [4,5]. This transition reflects a growing understanding that brands are not merely economic tools; they are also powerful communicative acts that engage with broader cultural and social discourses. By examining brands through the lens of discourse studies, researchers can explore how brands use language, symbols, and narratives to construct meaning and identities.

In an English global era, commercial branding is a key player in shaping identities at the crossroads of discourse and culture [6]. Street signs display commercial brands in more than one language especially where English and multilingualism are prevalent [5,7].

In Saudi Arabia, commercial branding involves a complex negotiation of cultural identity where the interplay of traditional and hybrid elements in food heritage creates a dynamic balance between diversity and integration, as examined by Greco [8] who investigated how Saudi cultural identity is constructed and debated amidst ongoing transformations, considering both gradual and sudden societal changes that influence the social environment and national image. The study considered whether these changes involve creating new identities or revitalizing existing ones. Language policy also gives priority to Arabic on shop signs; something that maintains national identity. In the meantime, millions of Muslims visit the country for Hajj and Umrah; something that makes Saudi Arabia a melting pot of cultures [9]. Millions of Muslims from all over the globe visit Mecca for pilgrimage, an annual event. Similarly, millions of Muslims come for Umrah during the year. These religious tourists come from different linguistic backgrounds including Arabic, Urdu, Malay, Persian, Spanish and English. Idris [10] pointed out that performing Hajj did not only strengthen the equality of Muslims in front of Allah, but it is a chance for multilingualism and multiculturalism along the hard journey. Kalender and Tari Kasnakoglu [11] found out that pilgrims’ identity is clearly shown in every group of Muslim pilgrims. During these religious journeys, many cultural practices are exchanged including those of drinking coffee.

Coffee is deeply rooted in the Saudi culture. Coffee dates back to the 15th century when it was first introduced by Yemeni traders [12]. Coffee quickly became popular among Saudi people, and it was soon being cultivated locally. The drink grew widely and became a popular social drink served during gatherings and ceremonies. Coffee also plays an essential role in social interactions. It is customary for hosts to serve coffee to their guests as a sign of hospitality. The preparation and serving of coffee are considered an art form, with specific rituals that must be followed. Arabica coffee is one of the most popular and widely consumed coffee varieties in Saudi Arabia. It is grown in various regions across the country, including the highlands of Asir, Jazan, and Al-Baha. The production of Arabica coffee has been steadily increasing over the years, with a focus on sustainable farming practices and modern processing techniques. Saudi coffee is a traditional coffee that is made with cardamom, saffron, and other spices. It is usually served in small cups with dates.

In recent years, the landscape of coffee consumption has evolved with a growing number of coffee shops emerging across the country. These modern coffee shops not only offer a contemporary take on coffee but also play a role in shaping national identity by blending traditional values with new social trends. In line with the Saudi 2030 vision, a Saudi coffee campaign was launched as an ideological strategy propagated by the Saudi government to foster national identity. In 2022, the Ministry of Culture devoted that year for promoting Saudi coffee to enhance national identity and boost international exports. Being a sample of hospitality, and connected with various Saudi traditions, there must be a study to focus on the construction of identities on street signs to exhibit such cultural heritage. The campaign also has offered us an occasion to study the competing discourses on coffee shop signage on the road to Mecca. In so doing, this article examines the coffee shops as a site where discourses can compete and complement each other and how this can be understood from an analysis of the signage on display.

Subsequently, this study analyzes coffee shop signage from a discourse perspective to reveal the construction of national identity via language choices. In so doing, this study developed a conceptual framework grounded on CDA [13,14] and Gorter’s model of multilingual inequalities in public spaces [7]. A linguistic [13] and multimodal [15] toolbox from critical discourse analysis (CDA) was used to analyze the data. Accordingly, the present study investigates the competing branding discourses associated with coffee on the multi/bilingual commercial signage of coffee shops on a commercial road from Najran to Mecca, Saudi Arabia. It specifically aims to address the following research questions:

1. What are the competing branding discourses displayed on the commercial signs on coffee shops on the road from Najran to Mecca?
2. How much space is given to national identity on these commercial signs?

## Literature review

This section reviews relevant studies on LL and the discourse of brands. It reviews the socio-cultural context of national identity and branding. It also develops a conceptual framework grounded on CDA and LL.

### Linguistic landscape

Linguistic Landscape (LL) is a relatively new field that studies among others unequal display of languages on signs spread in the public sphere. Gorter [7] proposed a model for inequalities on commercial signs. He stated that the center of attention in LL work is “language in the environment, words and images displayed and exposed in public spaces” (p. 4). Already Cenoz and Gorter [16] asserted in similar words that LL is about analyzing "languages in context by focusing on the written [and multimodal] information that is available on language signs in a specific area" (p. 67).

Seargeant and Giaxoglou [5] connected LL with discourse studies to show how public exhibitions of language and symbols play crucial roles in regulating and contesting politics, and how they evoke strong emotions. The authors stated that public signage is an important domain in which discourses compete, negotiate and interact. They further added that the connection between LL and discourse studies is “a step toward opening up the scope of this type of research” (p.321). They concluded that there is a great scope for LL to revisit including CDA an “area of study [that] will continue to flourish as an important window on the complex relationships between language, culture and society” (p.322).

Several studies on LL have been carried out. Lam and Graddol [17] investigated the vertical semiotics of a skyscraper in Hongkong to highlight the significance of verticality in landscape-related research. By focusing on the vertical dimension, the study reveals how it enriches our understanding of the interaction between discursive and material landscapes, particularly concerning social actors, practices, access, and space functions. The authors proposed an analytic model that treats vertical landscapes as active forces in sociolinguistic processes, rather than mere contextual backgrounds.

Buckingham and Al-Athwary [18] compared the use of English on commercial signs in the neighboring countries of Oman and Yemen, focusing on the capital cities of Muscat and Sana’a. Data were gathered from four commercial centers in each city, resulting in a corpus of respectively 698 and 393 signs. Their results showed that all signs featured both Arabic and English, but there were important differences in frequency and in content. Less than one percent of all signs contained other languages. In Muscat, both languages were given equal prominence through font size or color in rather predictable patterns of layout and language choice. In contrast, in Sana’a, most signs are in Arabic, where English appears in Arabic transliteration, especially in the names of establishments. The linguistic landscape reflects the different social functions of English and its relative position versus Arabic. In Muscat, English functions as a lingua franca, but in Sana´a English is used more for emblematic purposes, leading, for example, in Muscat to longer phrases in English and in Sana´a to individual words or use of the Arabic script for English words.

Buckingham [19] explored how the promotion of goods and services is linguistically expressed in commercial signs in shops in modern Omani society. The study analyzed linguistic characteristics using data from a collection of over 1,600 nationwide shop signs. The study identified processes like incorporating cultural concepts, introducing new words, borrowing from other languages, using foreign cultural references, and employing repetition, attributes, and specific strategies to make signs more noticeable and explicit. The study identified certain newly introduced words that are widely used across the country, implying their widespread acceptance and gradual adaptation within this context.

Alsaif and Starks [20] examined the linguistic landscape of the Grand Mosque in Mecca, a religious site. The authors could show that on the walls and ceilings, in the holiness domain, all verses are only written in Classical Arabic. In contrast, signs for locating purposes or wayfinding were mainly written in Modern Arabic and English, or, some became trilingual by adding Urdu. A number of electronic signs were used for education purposes or for giving further instructions to visitors. Those signs conveyed equivalent messages in multiple languages, adding among others French, Indonesian and Turkish to the repertoire. The authors emphasize the relation between the usage domain, the languages and the materials of the signs.

Some of the above studies add to our understanding of using English as a global language. Unlike Lam and Graddol [17], our present study is concerned with horizontal linguistic landscape of a road to Mecca.

### Toward discourse of brands

Existing approaches to branding defined it as a brand community associated with a geographical area [3]. However, Lupinek’s [21] review found that branding has expanded its boundaries in a global era. Acosta and Devasagayam [1] extended the notion of brand community to what they call brand cult. This latter concept, however, puts an emphasis only on the loyalty of a group to a brand; something that neglects the discursive construction of a brand as a name or sign that portrays identities.

In a study entitled "critical discourse studies and branding", Lischinsky [22] examined the various discursive features and strategies used to control the values and attributes associated with a brand. Lischinsky highlighted the significance of interpersonal features and their influence not only on consumers but also on other stakeholders. The author concluded that a comprehensive critical approach to branding discourse is still lacking and remains a topic for further investigation.

Seargeant and Giaxoglou [5] showed how public exhibitions of language and symbols play crucial roles in regulating and contesting politics, and how they evoke strong emotions. The authors stated that public signage is an important domain in which discourses compete, negotiate, and interact. They further added that the connection between public signage and discourse studies is “a step toward opening up the scope of this type of research” (p.321). They concluded that there is a great scope for public signage to be investigated from the perspective of CDA.

From a discourse perspective, brands on commercial signs can construct an ideational function. In so doing, they can serve competitive power relations, for example, between local and global identities. As discourses [5], commercial signs are essential components that construct competing identities. These competing discourses might appear in the form of local brands and/or global brands at the local scale of globalization [14]. In the context of globalization, local brands might be in a position of resistance. Therefore, this study employs a discourse perspective on brands.

### Studies on coffee branding

A few studies investigated global signage of coffee shops in Indonesia [23], in Colombia [24], in Taiwan [25], in Malaysia [26] and in Saudi Arabia [27]. These studies indicated that there is strong competition between local brands and global brands of coffee. This competition has manifested not only in coffee signage but also in adaptive cultural practices.

Purnomo et al. [23] investigated how Indonesia’s local coffee culture responds to the influence of global coffee trends introduced by *Starbucks* and similar modern coffee shops in the past decade. The research found that local coffee shops in Indonesia have incorporated some global elements while preserving their unique characteristics. Despite some resistance, this adaptation takes an encapsulation approach, where Western practices like product standardization and processing techniques are embraced, along with certain menu items.

Muniz Martinez [24] discussed the creation of a place brand for Colombia’s coffee region, emphasizing the shift from traditional marketing approaches led by a single institution toward a more modern concept of network branding involving multiple stakeholders. The region not only produces quality coffee but also promotes coffee-themed rural tourism to enhance its global economic and cultural positioning. The study explored the Coffee Triangle case, highlighting the complex interactions between various stakeholders and public and private institutions at diverse levels.

Wang et al. [28] investigated factors influencing Gen-Z consumers’ preferences for specialty coffee in Taiwan, focusing on gendered consumption, willingness to pay, and socio-cultural context. The study conducted interviews and taste tests to gather data from Gen-Z consumers. Findings reveal that specialty coffee is perceived as costly and not a frequent purchase among young consumers. However, it is associated with health benefits and serves as a status symbol, especially on social media platforms like Instagram and Facebook. The study also notes a gender effect, with females more inclined to consume specialty coffee than males.

Le et al. [26] investigated how young customers’ perceptions of the green image of trendy coffee cafes impact their environmental and product attitudes, and how these attitudes influence their citizenship behavior and intention to revisit the cafes. Data was collected from 207 young Malaysian cafe-goers. The study found that a green image enhances both environmental and product attitudes.

Thompson and Arsel [25] employed the term of the "hegemonic brandscape" to explore how the cultural influence of Starbucks shapes the socio-cultural environment of local coffee shops. The "Starbucks revolution" had set a standard for the ideal coffee shop experience, affecting local shops whether they positioned themselves as alternatives to or extensions of Starbucks. Local coffee shop culture offered access to cultural capital that can reduce class-based differences in aesthetic tastes.

Al-Hussami et al. [27] scrutinized signs of coffee shops in Abha, Saudi Arabia. The study highlighted that like other types of transformations, coffee has opened its doors to be served not only at houses but on the roads, streets, and shopping centers. It has become a great business for different companies that provide various favorite types. This study found Arabic or English monolingual signs, Arabic-English bilingual signs, and transliteration of English signs. This study examined a city where public signs are close to each other. It would be of great significance to examine another research site where public signs are scattered on a long road. Such distance is a great domain for the discursive construction of coffee branding.

In a word, coffee serves as a potent symbol for constructing national identity in Saudi Arabia, embodying values of hospitality, tradition, and unity. Its historical significance, cultural rituals, and contemporary relevance underscore its enduring importance in shaping societal norms and fostering a sense of belonging among people as well as internationalization. As Saudi Arabia navigates the complexities of modernization and globalization, coffee is still a steadfast emblem of cultural pride and heritage, serving as a source of identity in an ever-changing world.

### Discourses and identity on commercial signs

This study developed a conceptual framework grounded on CDA [13,14] and Gorter’s model of multilingual inequalities in public spaces [7].

CDA deals with the ideational function of language. In the same way, LL is concerned with the symbolic function of public signs. Both of them question the issue of power relations and construction of identities in a global era. Both of them aim to describe, analyze and explain inequalities disseminated in a text. A commercial sign is usually composed of words and images. It is also a matter of language choices and depends on its context [5]; something that aligns with CDA. Gorter and Cenoz [29] stated that names of commercial signs have cultural significance. English might be used for hegemony. Importantly, names have powerful discursive effect in social change.

CDA deals with three overlapping dimensions: text (description), discursive practice (interpretation), and sociocultural practice (explanation). The text dimension deals with linguistic choices. In discursive practice, ideological choices are part of revealing a discourse on commercial signs. This dimension involves both the production and consumption of a text or in our case the commercial signs. Sociocultural practice can be operationalized between discourses and identity. Chouliaraki and Fairclough [30] pointed out that one of the features of late modernity is the dialectic relationship between globalization and localization, between identity and difference. Such construction can be revealed by an integrated analysis for the languages and images of commercial signs. In general, commercial signs constitute a communicative event that mirrors the discursive and sociocultural practices in a particular society.

**Fig 1:**
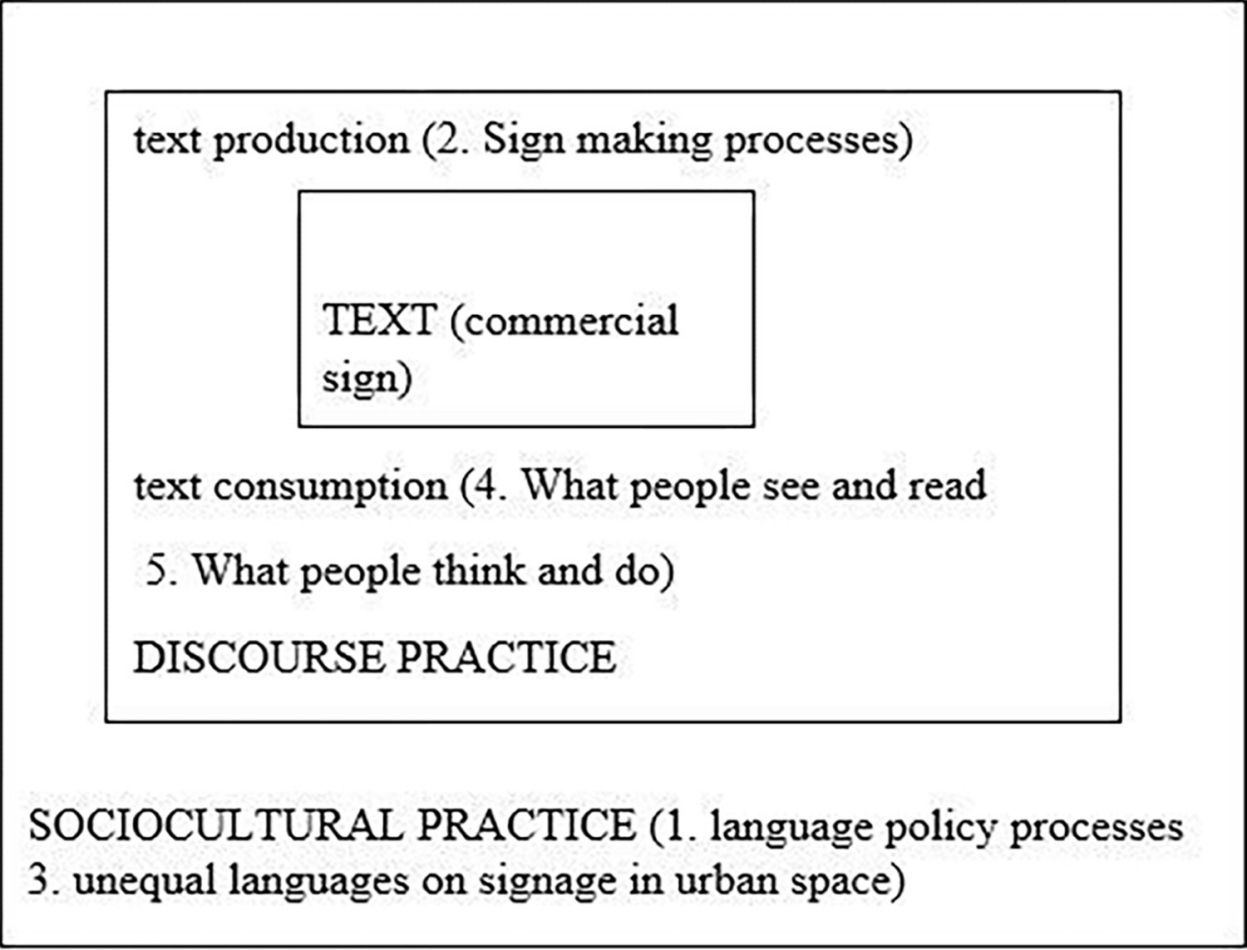
Conceptual framework for discourses and identity on commercial signs Adapted from Fairclough (12, p.59) and Gorter (16, p.19).

Similarly, Gorter [7] introduced a five component model that deals with inequality on commercial signs in a global era. The first component deals with processes related to language policy; something that can be associated with the third dimension of Fairclough’s CDA. The second component is concerned with the processes for production of signs. Sign producers can choose monolingual Arabic or English, bilingual, or multilingual names for commercial signs; something that can be linked with the second dimension of CDA. The third component is concerned with inequalities on signage. This component is close to the third dimension in CDA. The fourth and the fifth components are concerned about readers’ interpretations of commercial signs. These two components can be included within the second dimension of CDA. As a text, the sign is the object of analysis in Gorter’s model of inequalities. These five components can be operationalized with Fairclough’s three-dimensional approach for analyzing discourse and social change (Fig 1).

The analysis can be conducted on the language choices of commercial signs and their multimodal choices. For the text dimension, names on commercial signs are a matter of language choices: the choice to include Arabic, English and/or any other language. To name a coffee shop, producers of commercial signs might choose Arabic and/or English languages, depending on the shop owner’s interests. For example, in an Arabic context, an English-Arabic bilingual commercial sign might serve a prestigious global identity. This type of discursive construction can be revealed by analyzing the linguistic (word) and multimodal (image) choices of a commercial sign, the discursive practice, and the sociocultural practice. For the dimension of discursive practices, it involves processes for making and reading commercial signs. Sign producers can choose monolingual Arabic or English, bilingual, or multilingual names for commercial signs that target travelers. As for the third dimension, it takes into consideration issues of language policy, globalization, identities and inequalities.

Subsequently, this study employs this framework to examine coffee shop signage as a research site where discourses compete and how this competition can be understood from an analysis of the multilingual signage on display in a global context.

## Methods

### Research design

This study employs an empirical mixed-method research design which allows researchers to first quantify frequency and percentage of signs before moving to an in-depth qualitative CDA. This design is particularly suited to our study’s questions about the competing discourses associated with coffee on commercial signage. It reveals the prevalence and impact of multilingual commercial signs and the sociocultural dynamics influencing these signs.

For data collection, a driving approach and Google Maps are used to gather data on a road from Najran to Mecca, Saudi Arabia. The study uses CDA’s linguistic and multimodal analytical toolbox to interpret the symbolic function of the coffee shop signages. As empirical research, the CDA moved forth and back between data collection and the analytical toolbox. In reporting the findings, we presented the three levels of analysis as well as word and image analyses in an integrated way. To name a discourse, Fairclough [13] suggests bordering it with domain and perspective, e.g. English global discourse.

### Context of study

The Ministry of Culture of Saudi Arabia launched the initiative of 2022 as the year of Saudi coffee in line with the Saudi 2030 vision. The campaign aimed to raise awareness about locally produced coffee and its related culture, to increase its visibility, to strengthen national identity, and to enhance international exports. The campaign intended to promote the development of the local coffee industry, as well as encourage local coffee shops to show that they serve Saudi coffee. The Saudi Coffee 2022 campaign was an initiative that promoted the production of Saudi coffee and the use of coffee as an emblem of national identity.

A special logo was created for the government campaign, which emphasizes the visual identity of the Year of Saudi Coffee. The logo used an Arabic-English bilingual sign where Arabic was foregrounded and highlighted. It was inspired by the traditional “Al Finjan"; a type of cup made of mud, which has references related to drinking coffee and to Saudi values of generosity and hospitality. It was further inspired by the Saudi Arabian flag, which includes a palm tree and two swords. While the palm tree stands for dates, which are usually served with coffee, the two swords symbolize power.

Commercial signs on coffee shops are regulated under a Saudi royal decree published by the Ministry of Commerce. In this decree, Article no. 3 revealed that The trade name shall include Arabic or Arabized words and shall not include any foreign words; except for the names of foreign companies registered abroad, companies of well-known international names, and companies with joint (mixed) capital issued by a Decree of the Minister of Commerce (Article 3, the Law of Trade Names, p. 3).

These regulations stipulate the use of the Arabic language as it is the official and national language in Saudi Arabia. Foreignness is expressed with foreign words. Yet, there is an exception for the names of foreign companies, international names, and joint companies. While foreignness aligns with the concept of English as a foreign language, internationalization matches with English as a lingua franca. Article no. 12 also declared that "the merchant shall write his trade name on the front of his commercial shop” (Article 12, the Law of Trade Names, p. 4). This step regulates the process and identifies the extent to which names and signs comply with the ministry policies, and the purpose behind the policies, which would indicate what the government hopes to identify the extent to which the language policy was implemented on commercial signs.

### Data collection

This study collected coffee shops’ commercial signs on a road from Najran to Mecca. As a historical city, Najran used to be a major point of contact between the north and south of the Arabian Peninsula during the first millennium BC. Mecca is a religious destination that targets millions of travelers for Hajj and Umrah every year. On the road to Mecca, about 1000 kilometers, travelers need to stay for a break and drink coffee to be awake during their trips. This need created a great business opportunity for coffee shops that attract travelers through various names of coffee shops.

Three ways lead to Mecca, but this study selected the road along the coast as it crosses many regions and towns. The road crosses four regions: Najran, Asir, Jazan, and Mecca. In these regions, the journey passes some tourist cities and towns.

The journey took place during March 2 and 8, 2023. While driving and observing, the first author looked for coffee shops along the road. Upon locating a coffee shop, he slows down and asks his research assistant to take a photo(s) of the commercial signs of a coffee shop. There is at least one coffee shop at every petrol station. It seems that coffee shops near petrol stations are good businesses where travelers can stop and buy coffee.

These coffee shops are small cabins (about 15 square meters). They are designed in a drive-through way that provides fast coffee service. They have become increasingly popular in urban areas to attract both pedestrians and drivers. They provide both take-away and drive-through services, catering to the fast-paced lifestyle. The cabins’ menus feature a variety of coffee beverages, including espresso, lattes, cappuccinos, and cold brews, as well as teas. Despite their limited space, the layout and equipment of these cabins are optimized for operational efficiency, ensuring rapid service. The proliferation of these cabins reflects a broader trend towards convenience and efficiency in urban living, offering a quick and accessible solution for quality coffee on the go.

To ensure precise data collection, the research assistant initially compiled data from 45 commercial signs situated on both sides of the road. To validate the accuracy of the identified coffee shop names, these signs were employed as search keywords within the Google Maps platform for cross-verification. Duplicate entries of a coffee shop’s name were deliberately omitted upon discovery during the search process. Subsequently, an additional 43 distinct coffee shop names were procured through an exhaustive search on Google Maps. For instance, "Barn’s" was treated as one coffee shop, despite its operation of more than 170 establishments nationwide, with a substantial presence of at least 20 along the route to Mecca. Notably, for data compilation, a solitary photograph was employed to represent each coffee shop name, resulting in the documentation of a total of 88 distinct names.

### Data analysis

On the road to Mecca, the collected signs form a communicative event for quantitative and qualitative analyses. For quantitative analysis, all photographs of signs of coffee shops were coded in an Excel database. The coding scheme of the signs included categories such as the name of the coffee shop, the language(s) displayed (monolingual, bilingual, or multilingual), and a general description of each sign. An interpretation of each sign was given by providing a short description, and an explanation of the pattern encountered. The Excel database also contains multimodal features such as prominence (foregrounded, backgrounded, balanced), language directions (right/left or both), colors, font sizes (big, medium, small), and special semiotic remarks (such as including or excluding coffee cup, pot or dallah).

**Fig 2:**
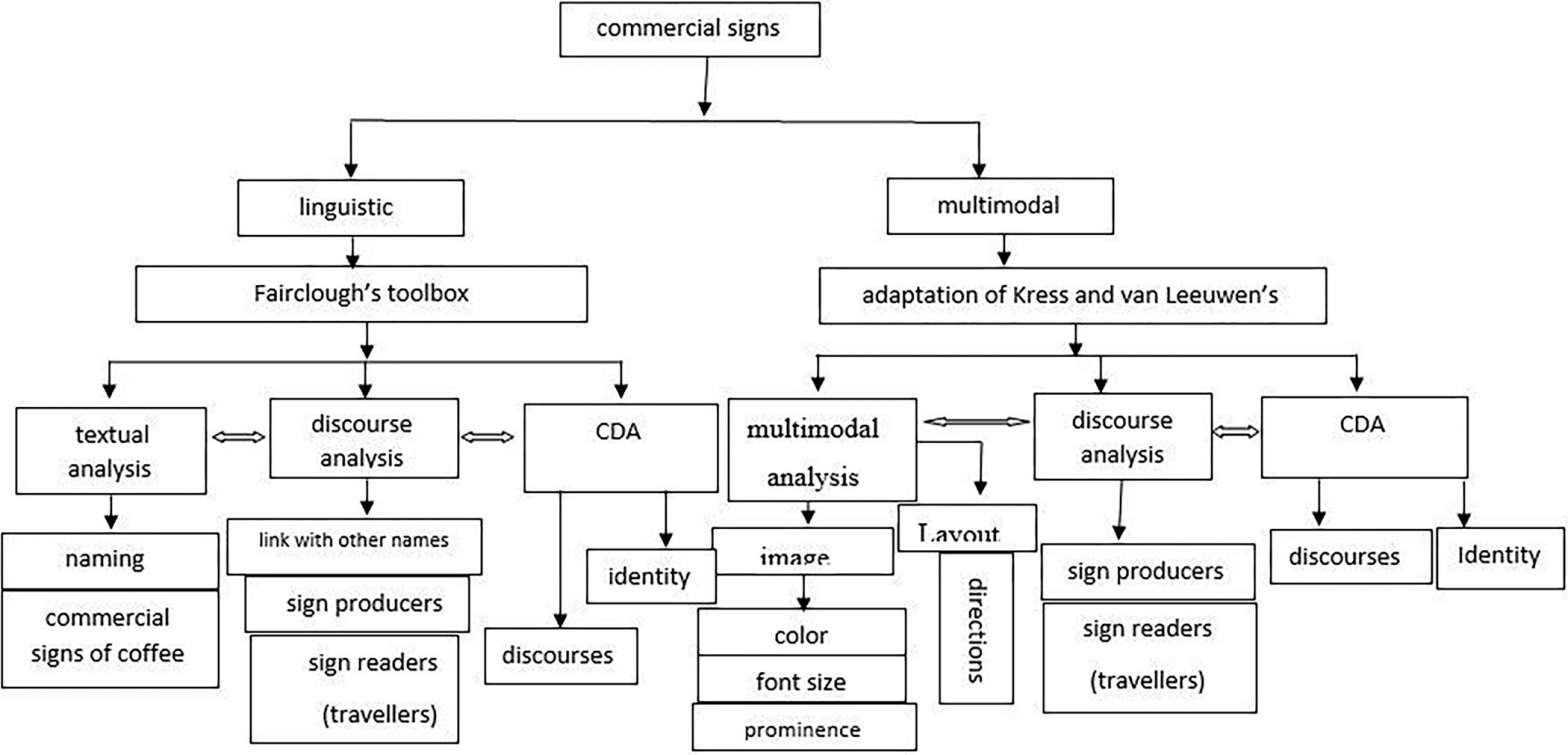
Analytical toolbox for analyzing competing branding discourses and identities on commercial signs.

For qualitative analysis, we adopted the three levels of analysis for words [13,31] and images [15]: textual analysis, discourse analysis, and CDA (Fig 2).

For textual analysis, we examined the brands on coffee shop signages including multilingual, bilingual, or monolingual choices: the choice to use English and/or Arabic language. For discourse analysis, we followed this division that producers of commercial signs might choose global or local brands depending on the shop owner’s interests. Coffee shop signs’ producers can choose monolingual Arabic or English, bilingual, or multilingual names for commercial signs. For the third level of analysis, i.e., CDA, we examined issues of power relations between the global and the local identities. The analysis extends to issues of image directions (left, right, center, top, down) and issues of prominence (foregrounding, backgrounding, including, and excluding). These multimodal features play a significant role in designing a commercial sign. Such choices might reflect the global or local identities that operate on a commercial sign. It is the role of the analyst to reveal these competing discourses and reveal the space given to national identity.

In monolingual signs, Arabic monolingual signs represent national identity. English monolingual signs represent global hegemonic discourse. In bilingual signs, however, the languages displayed included "Arabic-English" and "English-Arabic", depending on the source language on the sign, for which translation and transliteration were noted separately. When English is used as the source language it is considered a prestigious and or hegemonic language; something that reflects symbolic function, and when Arabic is used as the source language, English is considered an international language. This position is grounded on two perspectives about the use of English on public signs. On the one hand, using English rather than the national language on street signs is considered a kind of English homogeny [32]. On the other hand, English is used for internationalization. English can be used along with the national language for pragmatic purposes on street signs [25].

Commercial signs are analyzed through three integrated levels: sign analysis, discourse analysis and CDA. For sign analysis, words and images are analyzed. At this level, language choices can be in any language. It can be, for example, coffee (English), qahwa (Arabic) or kufi (transliteration). These names can be associated with local or global discourses. Similarly, local and global images can be associated with these words. The multimodal analysis also involves not only image choices but also color, font size, and prominence including issues of foregrounding or backgrounding local and global images. Discourse analysis involves the processes of sign production and the intended readers of these signs i.e. the travelers to Mecca, the link with other brands. CDA, the third level of analysis, depends on the context of the study. This study operationalized this level of analysis in the context of localization and globalization. According to CDA, these levels of analysis are evident in the world of the text or in our case, the world of commercial signs.

## Findings

Although the road to Mecca is a route for Muslim pilgrimages from distinct parts of the world, no multilingual signs (including Urdu or Malay) are found on the commercial signs of coffee shops. This practice could be attributed to the effect of English where only English has its co-existence with the Arabic language.

Table 1 shows 88 different monolingual and bilingual names of coffee shops on the road to Mecca.

**Table 1:** Language use on names of coffee shops signs on the road to Mecca.

Multilingualism	Monolingual		Bilingual		Total
Languages	Arabic	English	Arabic-English	English-Arabic	
Frequency	37	7	14	30	88
Percentages	42% local	8% global	16% A-E glocal	34% E-A glocal	100%
Orientation	national identity	globalization	national identity	globalization	

As Table 1 shows half of the signs are glocal bilingual brands. However, the source of two-thirds (34%) of these brands is the English language. The source of 16% of these signs is Arabic. When it comes to monolingual signs, 42% Arabic monolingual signs represent local brands, and 8% English monolingual signs portray global brands of coffee shops on the road to Mecca. 58% of the signs give a substantial space to Arabic national identity in Arabic monolingual signs and Arabic-English bilingual signs.

In line with the research questions, the findings are presented in the next four sub-sections. For the first research question, the study reports samples of English-Arabic glocal discourse and English global discourse. For the second research question, the study presents samples for Arabic local discourse and Arabic-English glocal discourse.

### English-Arabic glocal discourse

**Fig 3:**
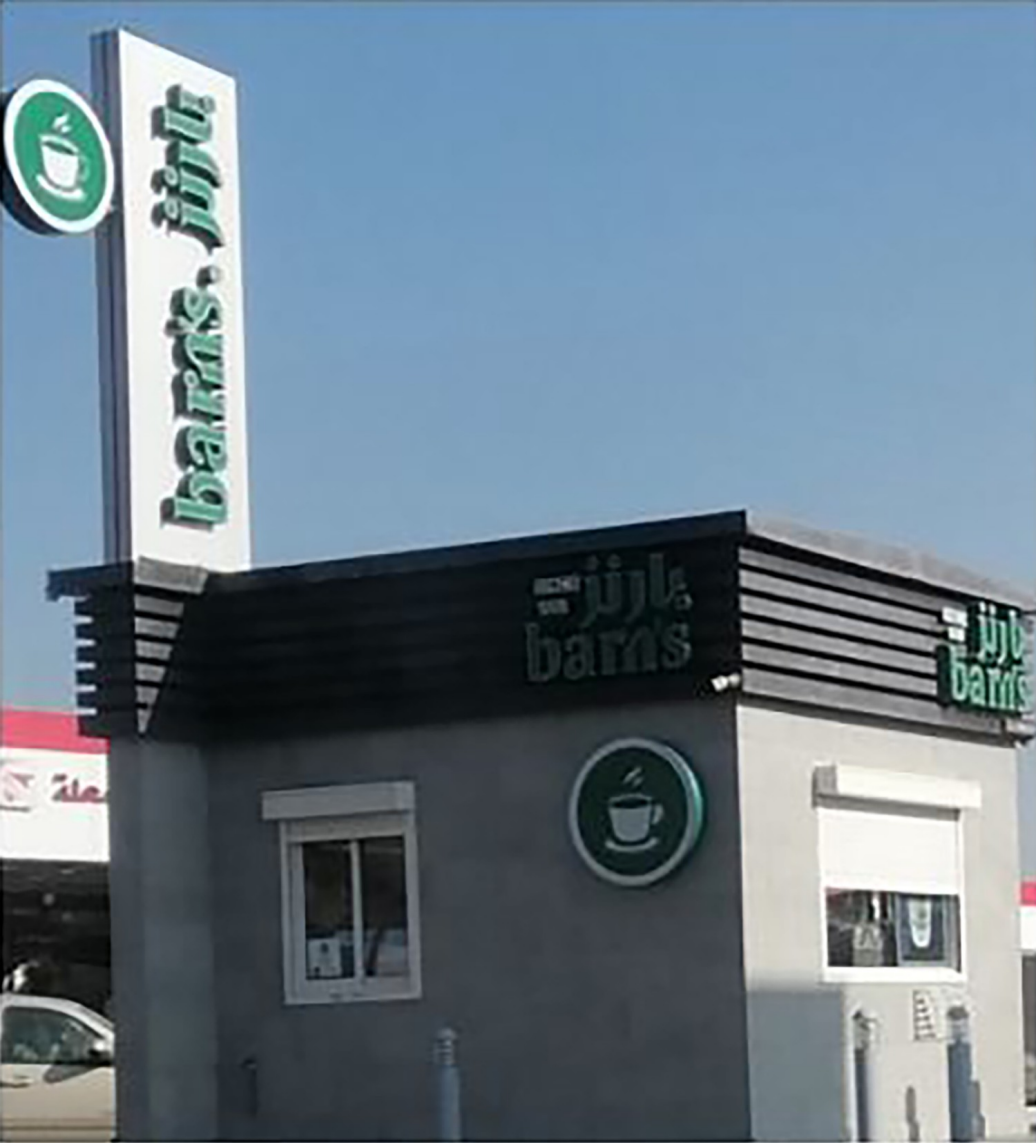
Sample of English-Arabic glocal discourse.

Thirty English-Arabic bilingual signs portray glocal discourse./ kufi/, transliteration of the word coffee, is usually associated with this discourse; something that serves a hegemonic function [25]. English is foregrounded as the source language. For example, one can come across the sign of *barn’s* (Fig 3) in at least twenty places.

This bilingual name projects a glocal discourse not only in its name, but its multimodal features also indicate a glocal orientation. The sign foregrounds the transliteration of the English word *barn’s* on top. The use of the green color completely matches the color of the worldwide *Starbucks* chain. There seems to be an added national or local dimension in the choice of the small white cup as part of the sign. Such a choice might serve as a promotional sign to attract local customers to the small white cups that they used to drink from at home. However, the coffee offered in this shop is not served in the small cups shown on the sign.

**Fig 4:**
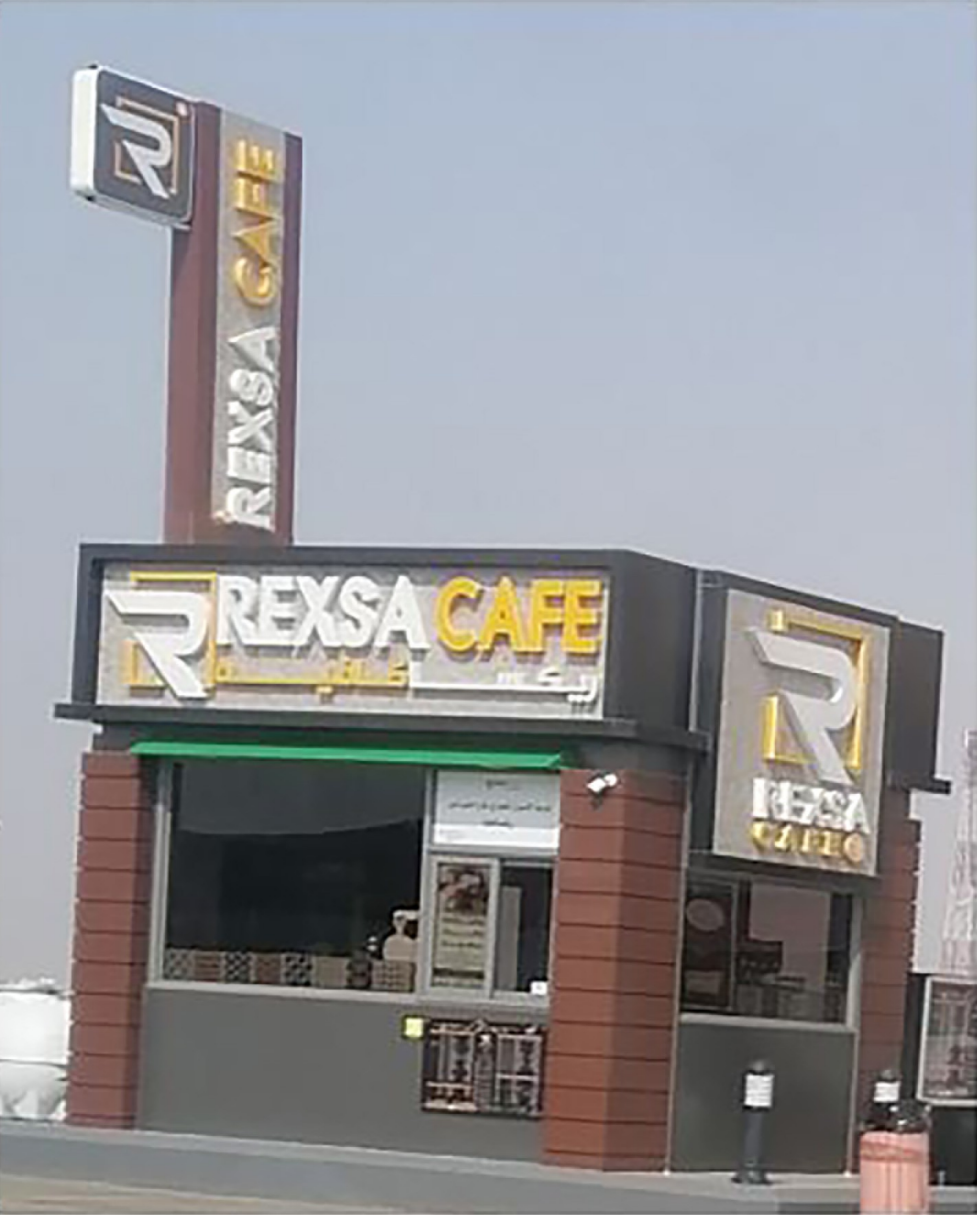
Sample of English-Arabic glocal discourse.

Another example of English-Arabic bilingual signs is REXSA Café which constructs a glocal brand (Fig 4). This glocal discourse is manifested in the use of English as well as Arabic transliteration.

The brand seems to be a blended name of REX which means King and SA which might stand for Saudi Arabia. Although this brand employs local meaning it uses English and Arabic transliterated words. Local travelers would not be able to understand the meaning of REXSA. This is because they might be unfamiliar with such an English blended name and its transliteration into Arabic. It is by the passage of time that travelers get used to the brand where there are 27 branches, according to its website, most of them are on the road to Mecca. Similarly, international travelers who pass the road would be familiar with the brand through such discursive practice. However, for global readers, the sexual overtone of "REXSA Café" hinges on the phonetic resemblance between "rex" and "sex." While phonetic associations can indeed influence customers’ perception, it is essential to consider contextual factors and cultural nuances. The link between "rex" and "sex" may not be universally perceived, as semantic interpretations are contingent upon individual experiences and cultural backgrounds. In this way, REXSA establishes its glocal discourse, at the local scale of globalization, by using English as a prestigious language.

The multimodal analysis shows that this sign foregrounds its brand, REXSA. Its brand appears to be a half-white capital R surrounded by yellow lines that form a square. REXSA uses the white color and CAFÉ uses the yellow color. These colors substitute the Saudi yellow coffee served in a white cup. The three corners of the coffee shop use the brown color. A temporary announcement appears in Arabic. The English language is foregrounded in its use of capital letters, its direction, and its prominence. The English writing system (left to right) and bottom-up are used in this commercial sign. On the other hand, Arabic transliterated letters are backgrounded.

### Global english discourse

Seven English monolingual signs portrayed global branding discourse. These are TAXICAFE, Coffee 70% 30, NAY Coffee, WHIFF COFFEE, and X7 Coffee. English is foregrounded as the sole language.

**Fig 5:**
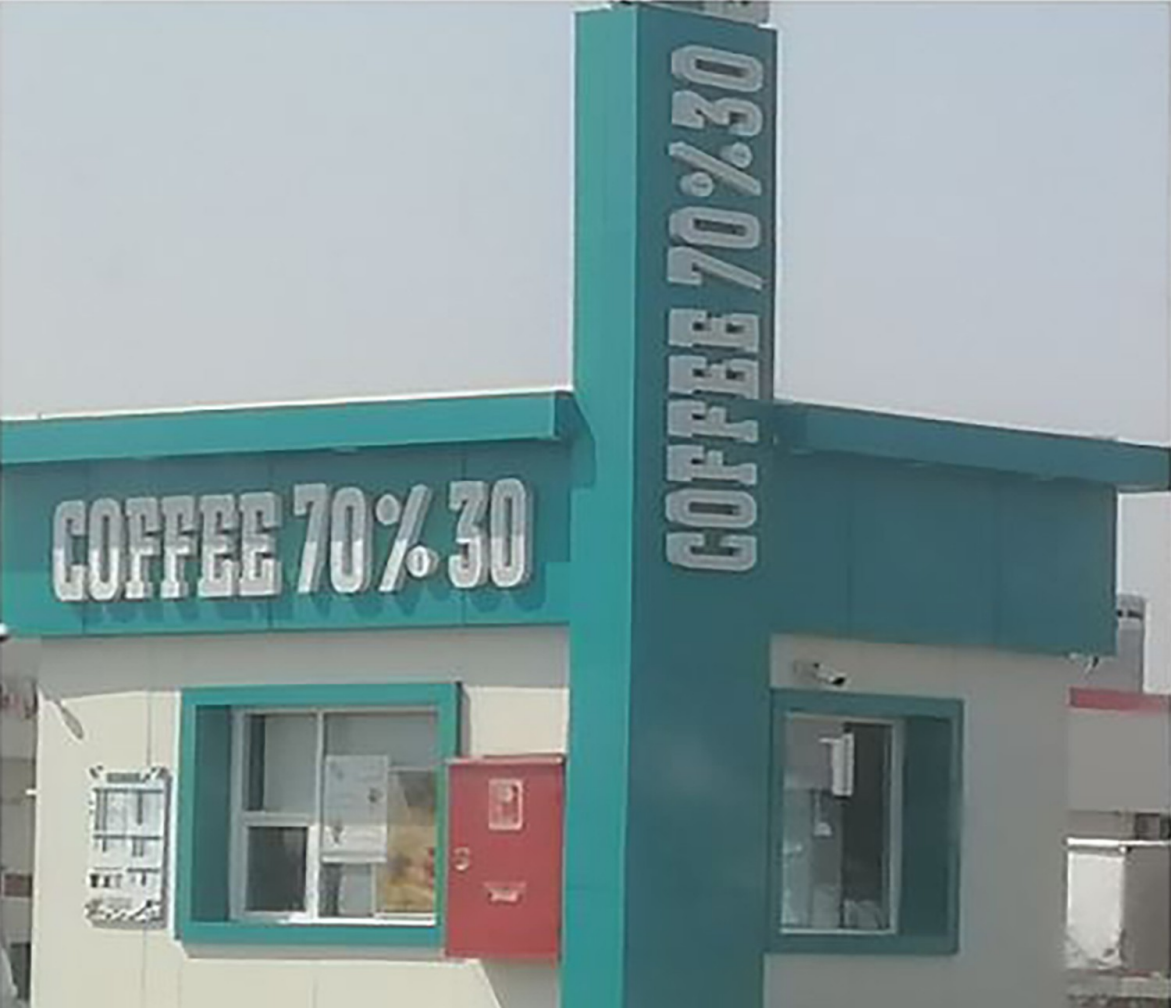
Sample of English global discourse.

For example, "Coffee 70% 30" (Fig 5) appears to be a linguistic shorthand that incorporates numerical elements to convey specific details about the coffee blend of 70% Arabica and 30% Robusta beans. The multimodal aspects, including visual and design elements, play a crucial role in enhancing global brand and consumer perception. Other manifestions of globalization are KAVD CAFÉ and CAFEE Q70. Although KAVD is not a standard English word, its use of Latin alphabets represents a global code. Similarly, the word CAFÉ is a French loanword, it is commonly used in English. Additionally, CAFEE appears to be a variant spelling of CAFÉ.

### Arabic local discourse

**Fig 6:**
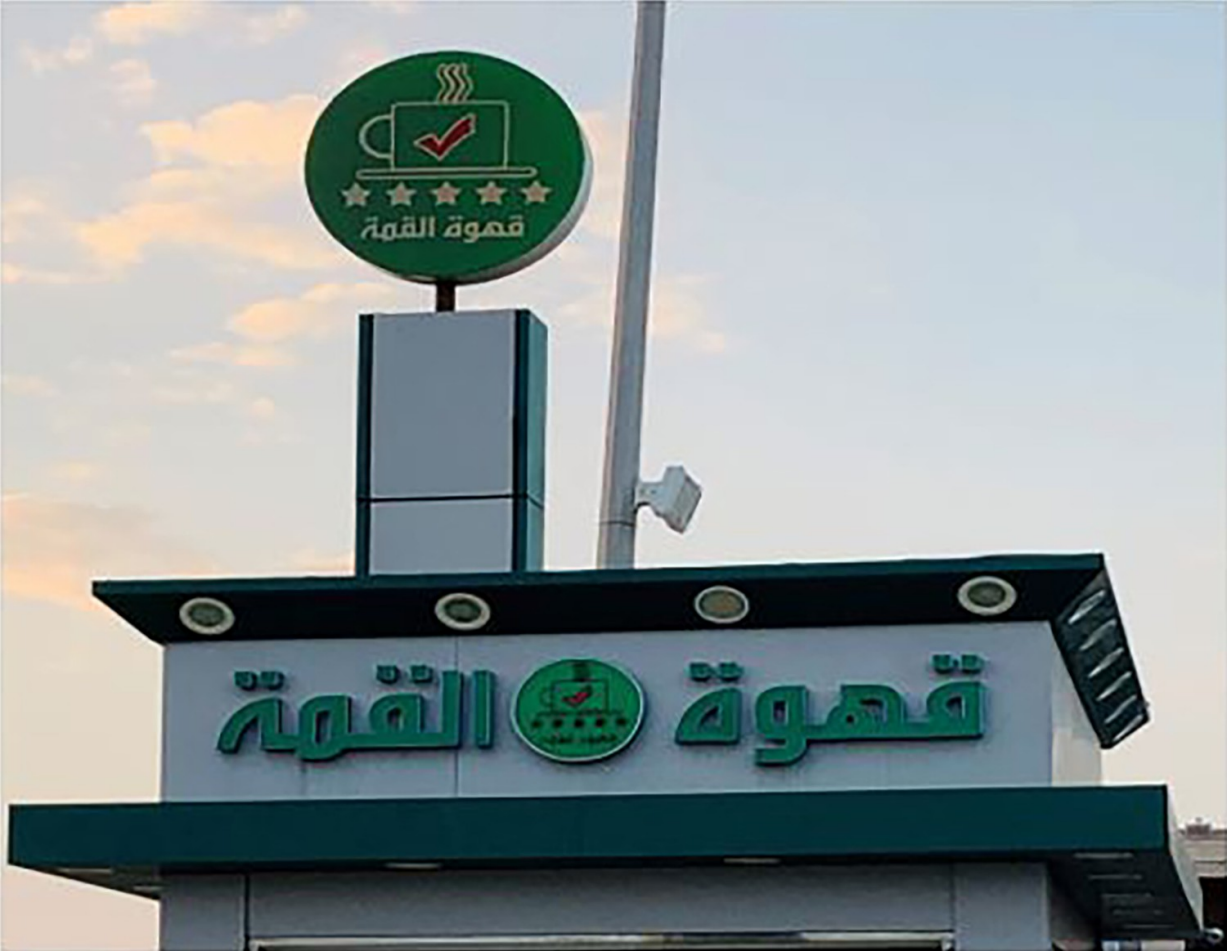
Sample of Arabic local discourse.

Thirty-seven Arabic monolingual signs portray local branding discourse. Arabic is foregrounded as the sole language. For example, one of the Arabic monolingual coffee shops is named /qahwat al-qimmah / which can be translated as Top Coffee (Fig 6).

This sign provides essential information about the product or brand being displayed. In this context, it may suggest that *Top Coffee* is a trusted and dependable brand. The presence of five stars is a common visual indicator of quality or excellence. It implies that *Top Coffee* is of high quality and has received top ratings or reviews. Such a choice can help attract customers looking for a premium coffee experience. The cup with a red tick mark implies that coffee meets certain standards or specifications, assuring customers of its quality. Overall, this commercial sign appears to be using a combination of color, text, and symbols to convey the message that *Top Coffee* is a high-quality and reliable coffee brand. The use of Arabic script also suggests that the target audience is Arabic-speaking travelers on the road to Mecca, which is a significant pilgrimage destination for Muslims. The sign aims to capture the attention of potential customers and communicate the brand’s value and quality.

### Arabic-English glocal discourse

**Fig 7:**
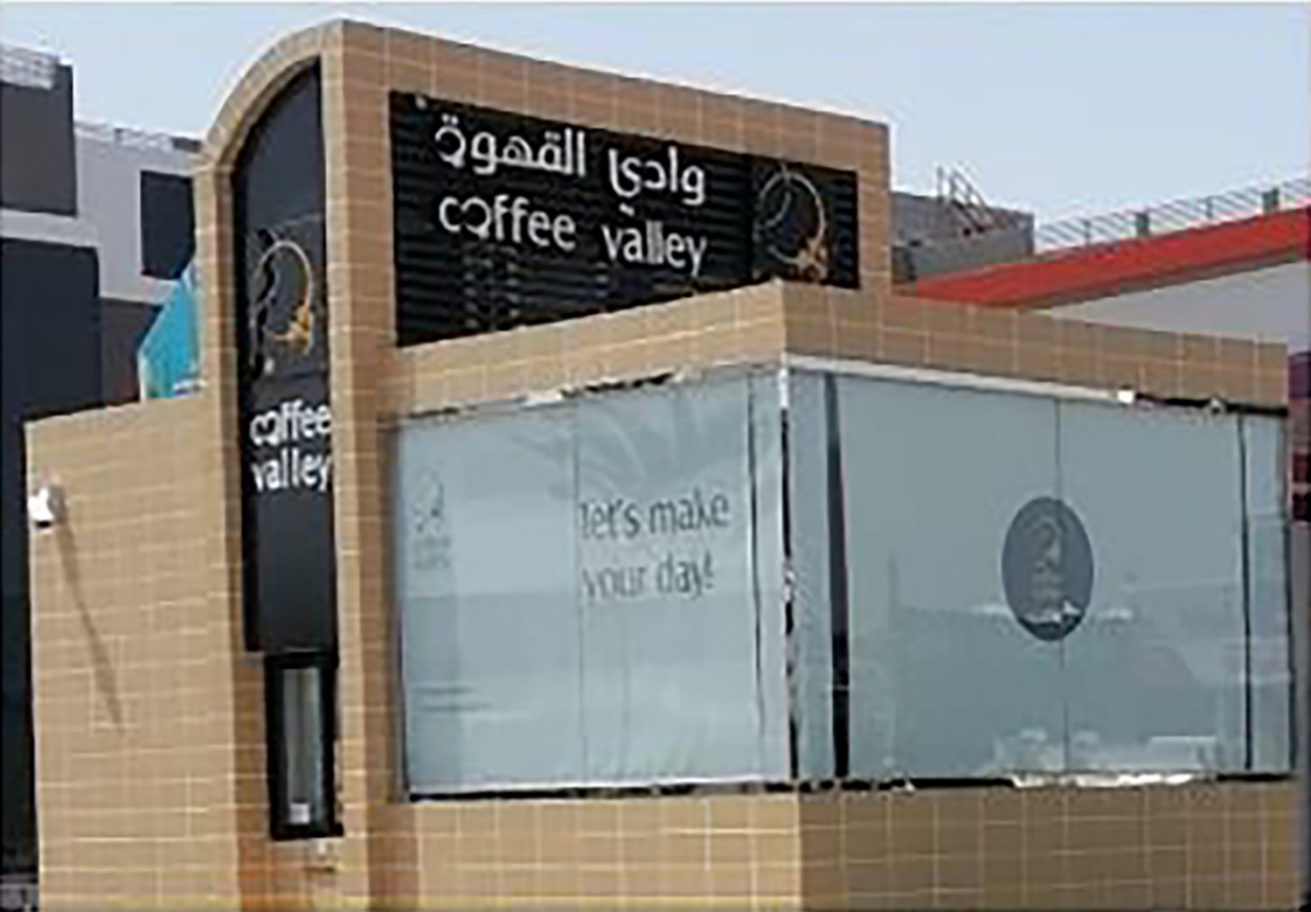
Sample of Arabic-English glocal discourse.

Fourteen Arabic-English bilingual signs portray Arabic-English glocal brands. Arabic is foregrounded as the source language. For example, /wadi al-qahwa/ *Coffee Valley* is an Arabic-English bilingual sign (Fig 7).

This sign translates the Arabic word /qahwa/ into English coffee. It uses both the Arabic word /وادي/ and the English valley. The word coffee is associated with valley. Arabic name is also foregrounded on top while the English translation was inserted below the Arabic name. Although the cup is not associated with a brand name, some multimodal features build a glocal discourse such as the color of the building blocks which matches the color of sand that reflects a desert discourse; something that adds to Saudi desert identity.

## Discussion

**Fig 8:**
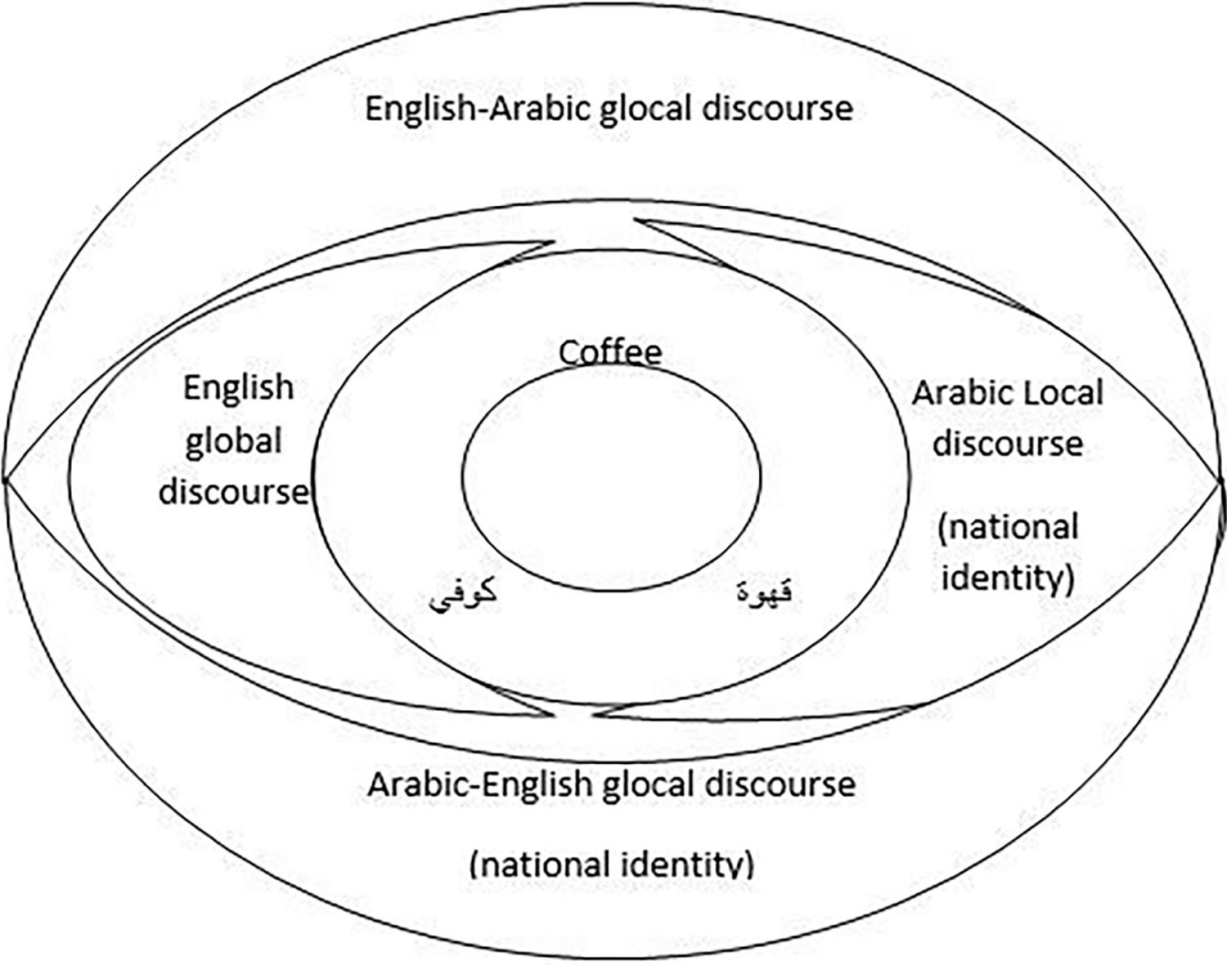
Competing discourses of coffee branding.

In line with the two research questions, the findings are discussed. The findings reveal three names of coffee used on the road to Mecca:/ qahwa/, coffee, and kufi/ /. These names are associated with four competitive discourses: namely, English-Arabic glocal discourse, English global discourse, Arabic local discourse, and Arabic-English glocal discourse (Fig 8).

Coffee (English) and /qahwa/(Arabic) can be found in the English and Arabic dictionaries. However, the word kufi/ / is a transliteration in Arabic for the word /coffee/. The word /kafiah/ is a naturalization for the word /café/. These Arabicized transliterated names are neither Arabic nor English; they have English meanings in Arabic forms. Their Arabic forms serve prestigious English function. Although these forms cannot be found in an Arabic dictionary, their use coincides with the notion of discursive construction [33] of branding; it indicates that these new forms might be added to an Arabic dictionary over time.

### Global branding discourses

English-Arabic glocal discourse is portrayed on two-thirds of the bilingual commercial signs where English is the source language. This finding coincides with several studies that deal with English as a hegemonic language. Purnomo’s et al. [23] revealed that Western coffee practices localized their development and advocated for fair business practices in Indonesia. Similarly, Thompson and Arsel [25] found that *Starbucks* influenced the socio-cultural environment of local coffee shops, leading to two types of anti-Starbucks clients with diverse preferences. Equally, Rubdy [34] found a third space that does not align with being purely Hindi or English, traditional or modern, indigenous, or cosmopolitan. Pošeiko [4] reported similar glocal commercial names in the LL of the Baltic States. Laihonen [35] also emphasized the intricate interaction of historical, legal, and cultural factors impacting language use in commercial signage, particularly the challenges for local shop owners in balancing local and global identities. This aligns with our study, suggesting a partial agreement, as we observe the dominance of glocal branding in the signage of coffee shops in Saudi Arabia.

English global discourse is also associated with the English monolingual use of coffee in the names of a few coffee shops on the commercial signs. The use of English projects global branding. At the same time, it constructs a hegemonic discourse for companies that transcend national borders. The recognition of global branding discourse in the use of English on commercial signs corresponds with Rababah et al. [32] who found a pivotal role of English in projecting a global discourse and participating in global branding practices. Similarly, Thompson and Arsel [25] reported the hegemonic brandscape of *Starbucks* and its impact on the coffee shop culture.

Other global brands appear in the form of French origin. This finding is in agreement with Buckingham [19,36] who found that borrowing from other languages and using foreign cultural references is one of the global manfiestions of the LL in Oman. These manifestions of globalization also coincide with Ben-Rafael and Ben-Rafael’s [37] global code of big commercial names that can be found around the world.

These identified discourses resonate with Buckingham’s [19,36] and Buckingham and Al-Athwary [18] emphasis on the lexicalization of cultural concepts and the utilization of structural features in the LL. Similarly, Purnomo et al. [6] showed that Western coffee culture transformed Indonesian coffee culture among the middle class. This transformation involved a nuanced response, blending elements from both cultures while maintaining distinct boundaries. Indonesia’s evolving coffee culture stands out with its "cultural encapsulation process" and emphasis on fairness and identity.

### Space for national identity

When it comes to the space given to national identity, one can find substantial space for national identity in the Arabic local discourse and the Arabic-English glocal discourse. National identity has substantial space in the Arabic monolingual signs and one-third of the bilingual signs where Arabic is the source language. National identity is represented in monolingual Arabic signs. It is also manifested in bilingual signs. The study interprets the coffee shop signs where Arabic is used solely as monolingual and bilingual (Arabic-English). Arabic local discourse is associated with Arabic monolingual use of /qahwa / and / kufi/. The study revealed that Arabic as the official language is used in 42% of the coffee shop signs. This confirms the Saudi authority’s tendency to safeguard the national identity in a world characterized by quick transformation toward globalization.

However, the use of transliteration of coffee coincides with Al-Hussami et al.’s [27] findings that highlighted how coffee practices in Abha city have changed over time like other social changes. This transformation of Saudi society has created a completely new coffee scene, and the outdoor signage of coffee shops and cabins can reflect these social changes.

Now we come into the use of Arabic along with English in coffee signs. Arabic- English glocal discourse is manifested in the names of coffee shops on one-third of the bilingual commercial signs where Arabic is the source language. The study reported that 14% of coffee signs were Arabic-English oriented. To calculate the 42% where Arabic is used as a sole local language and 14% where Arabic is used as bilingual (source) language, we reach 56%. This shows that Arabic exceeds English. This manifestation of discourse is expanding in line with the Saudi 2030 vision that supports its own identity while internationalization. This discourse coincides with the 2022 Year of Saudi Coffee campaign promoted by the Ministry of Culture in Saudi Arabia to strengthen national identity. The campaign featured a logo inspired by traditional coffee cups (Al Finjan) and the Saudi flag, symbolizing hospitality, generosity, and national pride. It used an Arabic-English bilingual sign in its logo where Arabic language is foregrounded as the source language. In that logo, Arabic language (qahwa) is foregrounded as the source language while English (coffee) is used for internationalization. The use of English in this logo indicates an international orientation for Saudi policy of branding. Recently, a new glocal brand has emerged. Its name is Jazean; it derives its name from Jazan; the first city producer of Arabica coffee in Saudi Arabia. Like Buckingham [19,36] and Buckingham and, Al-Athwary [18], this study found the prevalence of bilingual naming, which emphasized cultural identity and a resistance to homogenization or a desire for unique, localized experiences. Unlike these findings, coffee is considered a minor factor in the development of Brazil’s national identity [38].

Two important issues need further consideration. First, the widespread transliteration /kufi/ in Arabic form is used to serve a prestigious English function. Such use indicates a transformation in the use of the Arabic word /qahwa/. Second, the English language is foregrounded as a source language in two-thirds of the bilingual signs. This use indicates a global hegemony of the English language at the local scale of globalization; something that aligns with the conception of think globally and act locally. However, there is a need to move towards a notion of thinking and acting glocally. This is because the local/ national needs to be the source of identity that competes with the global. One needs to remember that the global is another local in its context. In other words, national identity has the right to employ its local discourse outside the local boundaries.

## Conclusion

This study investigates the competing discourses as well as the space given to national identity associated with coffee signage on the commercial signs of coffee shops on a road to Mecca, Saudi Arabia. It is found that Arabic local discourse, English-Arabic glocal discourse, Arabic-English glocal discourse, and English global discourse are associated with coffee. These competing discourses construct coffee branding on the research site. However, the use of the transliteration of /kufi/ in Arabic and the use of English as a source language in many bilingual signs constructs discursive hegemony. As for the space for national identity, Arabic monolingual, and Arabic-English bilingual local branding discourse give substantial space for national identity.

The study suggests some cultural and pedagogical implications for authorities and for bilingual teaching and learning. Officially, governments need to activate their language policies on commercial signs. Although the rules are regulations are well-established, there is a need to match the rules with the common practices in the commercial sectors. For sign production, commercial signs can be produced under the supervision of authorities and well-trained translators. Governments can adopt policies that affect coffee shop branding in terms of identity and advertising restrictions. Local coffee shops can effectively use branding to compete with larger global chains. Pedagogically, bilingual signs can be used as learning materials to enhance language awareness among students about the role of language in the discursive construction of identities. Bilingual instructors can encourage students to go beyond classroom boundaries and textbooks to question language choices around them. Public signage is a rich domain for bilingual students, instructors, and action researchers to examine language choices. Translation students can collect data for their research group projects to address translation issues, practices, and pragmatic errors.

This study has some limitations. The study dealt with commercial signs on a road to Mecca. The findings are limited to this communicative event and cannot be generalized. Another limitation is that the authors are bilingual in Arabic and English languages and cultures. They have done their best to avoid subjectivity in this interpretative nature of discourse analysis.

The discourse of coffee shop signage is a complex and multifaceted topic. Further research could explore coffee shop digital signage or billboard conveying meanings across various cultures. Understanding the semiotics and symbolism behind these signs can shed light on their impact on customers’ perceptions. Investigating the linguistic aspects of coffee shop signage can provide insights into how language choices affect customer experiences. Future studies could also focus on how coffee shops adapt their signage to local contexts while maintaining a global appeal. Exploring how glocal discourses emerge in diverse cultural settings can contribute to understanding the dynamics of globalization and localization. Other studies could employ this framework to investigate the discursive construction of other products and brands such as restaurants. A survey could examine how such discourses impact consumer behavior, choices, and preferences. One can compare cross-cultural branding and strategies for specific markets, brand loyalty, and identity.

## Supporting information

S1 File (DOCX)
