# Supplementary material for: On the road to Mecca: Branding discourses and national identity on coffee shop signage
Source: PLoS One. 2025 Feb 4;20(2):e0309829. doi: 10.1371/journal.pone.0309829 (PMC11793779; doi:10.1371/journal.pone.0309829)
Supplement: S1 File — (DOCX) [file pone.0309829.s001.docx]

**Data Availability**

| **Name of coffee shop sign**  **// indicates that Arabic letters have Romanized** | **Source of sign** |
| --- | --- |
| / Kofista/ | smartphone camera while smartphone camera while driving |
| / Moka Yashino/ | Google Map |
| / Lynn Kofiyeh /Leen Coffee | smartphone camera while driving |
| Repose Café / Rebus Café/ | smartphone camera while driving |
| GPR1 Café / JBR 1 Café/ | smartphone camera while driving |
| Brownies / Bravo Niz/ | smartphone camera while driving |
| / Kufi Haus /Coffee House | smartphone camera while driving |
| Coffee Cup / Kufi Kb/ | smartphone camera while driving |
| VAVA CUP / Jafi Kb/ | smartphone camera while driving |
| EVAD COFFEE / Iifad Café/ | smartphone camera while driving |
| Royal /Royal Café/ | smartphone camera while driving |
| Express Cup / Express Kb/ | smartphone camera while driving |
| Promo Café / Promo Café/ | smartphone camera while driving |
| White Coffee / Wite Café/ | smartphone camera while driving |
| / Nastretto Kofi / Nestereto Coffee | smartphone camera while driving |
| dip pie / Deep Bay/ | smartphone camera while driving |
| Barista / Barista/ | Google Map |
| Every Day Coffee / Kul Yawm/ | Google Map |
| eli / Eli/ | Google Map |
| / Aleef Cafe / ALEV CAFÉ | Google Map |
| YANA Coffee /Yana/ | Google Map |
| / Kandle / Candle | Google Map |
| Jailan /lalakhoua al-mukhata/ | Google Map |
| PanCo /banco/ | Google Map |
| / onza / Onza | Google Map |
| / rell kafia / RealCafe | Google Map |
| BEANS / bains/ | Google Map |
| Barn's (since 1992) / barnes/ | smartphone camera while driving |
| TAXICAFE | smartphone camera while driving |
| Coffee 70% 30 | smartphone camera while driving |
| NAY Coffee | Google Map |
| WHIFF COFFEE | Google Map |
| Jouden / gooden/ | Google Map |
| KAVD CAFÉ | Google Map |
| X7 Coffee | Google Map |
| CAFEE Q70 | Google Map |
| / maimon café/ Maymoun Cafe | smartphone camera while driving |
| / al-qahwah al-amrikiya/ | Google Map |
| / mughi moown latkadim mashroubat/ Moon Café | smartphone camera while driving |
| / coffi centre mentada/ | smartphone camera while driving |
| / caffi opshen/ | Google Map |
| / bernamj coffi/ | smartphone camera while driving |
| /coffi hale/ | smartphone camera while driving |
| /coffi tassalli/ | smartphone camera while driving |
| /coffi zen/ | smartphone camera while driving |
| /jamaneh coffi/ | smartphone camera while driving |
| /zad café/ | smartphone camera while driving |
| /coffi centre al-dawha/ | smartphone camera while driving |
| /coffi ishak qamar/ | smartphone camera while driving |
| /rakan round diwania/ | Google Map |
| /mughi al-rayef wan/ | Google Map |
| / tie coffi/ Tea Coffee | smartphone camera while driving |
| REXSA CAFÉ / rexha café/ | smartphone camera while driving |
| Café Knan / coffi kanan/ | smartphone camera while driving |
| / wadi al-qahwah/ Coffee Valley | smartphone camera while driving |
| Coffee / kahwa mukhtasa/ | Google Map |
| / rakan al-qahwah / Coffee Corner | smartphone camera while driving |
| / petelle/ Battela | smartphone camera while driving |
| KYAN / kian/ | smartphone camera while driving |
| / asl al-ban/ Coffee Origin | smartphone camera while driving |
| / kahwa/ Café | smartphone camera while driving |
| / kahwa al-tasia/ Nine Café | Google Map |
| LOUZ / luz/ | Google Map |
| / kahwa el-kaif/ Alkaiff Coffee | Google Map |
| / mughi diwania al-taj/ | smartphone camera while driving |
| / dalla al-assala lalakhoua al-arbia/ | smartphone camera while driving |
| /kahwa al-qama/ | smartphone camera while driving |
| /mughi mazag al-badiya/ | smartphone camera while driving |
| /mugha ralph al-shati/ | smartphone camera while driving |
| /madak al-qahwah/ | smartphone camera while driving |
| /mazag Moghrabi/ | smartphone camera while driving |
| /mughi eke/ | Google Map |
| /mughi al-muwadi/ | Google Map |
| /mughi al-almas al-aswad/ | Google Map |
| /mughi ola kivk/ | Google Map |
| /al-madhaq al-maghrebi/ | Google Map |
| /mughi al-juzeira/ | Google Map |
| / asrar al-ban/ Asrar Albon | Google Map |
| / diwania al-amada/ | Google Map |
| diwania rawai al-qahwah al-arbia | Google Map |
| / diwania al-dana/ Al-Dana Dewaniah / alakhoua al-arbia wallacklat chaabi/ | Google Map |
| /mentzah oumqhi Haithem/ | Google Map |
| /diwania al-assala/ | Google Map |
| /diwania al-mujallis/ | Google Map |
| / diwania al-saraya lalakhoua al-arbia/ | Google Map |
| / mughi al-wajha al-bahriye/ | Google Map |
| / dhuqi al-ali/ | Google Map |
| / diwania al-dyafa/ | Google Map |
| **Total** | **88** |
